# CCL18 synergises with high concentrations of glucose in stimulating fibronectin production in human renal tubuloepithelial cells

**DOI:** 10.1186/s12882-016-0352-1

**Published:** 2016-09-29

**Authors:** Rosa M. Montero, Gurjeet Bhangal, Charles D. Pusey, Andrew H. Frankel, Frederick W. K. Tam

**Affiliations:** Renal and Vascular Inflammation Section, Department of Medicine, Imperial College London, Hammersmith Hospital, London, W12 0NN UK

**Keywords:** CCL18, MCP-1, Fibronectin, Diabetic nephropathy, HK-2 cells

## Abstract

**Background:**

Diabetic nephropathy is the leading cause of end stage kidney disease worldwide. The pathogenesis of this disease remains elusive and multiple factors have been implicated. These include the effects of hyperglycaemia, haemodynamic and metabolic factors, and an inflammatory process that stimulates cellular signalling pathways leading to disease progression and severe fibrosis. Fibronectin (Fn) is an important protein of the extracellular matrix that is essential in fibrosis and its presence in increased amounts has been identified in the kidney in diabetic nephropathy.

**Methods:**

Proximal tubuloepithelial (HK-2) cells were stimulated with high glucose (30 mM D-glucose) or glycated albumin (500 μg/mmol) + 4 mM D-glucose or their controls, Mannitol (26 mM + 4 mM D-glucose) and 4 mM D-glucose, respectively. Following 48 h of stimulation the supernatant was collected and MTT [3-(4,5-dimethylthiazole-2,5-diphenyltetrazolium bromide] assay performed to assess cell viability. HK-2 cells were also stimulated in the above environments with recombinant CCL18 (rCCL18) or MCP-1 (rMCP-1) for 48 h with quantification of Fn levels using ELISA.

**Results:**

Co-stimulation of HK-2 cells with high concentrations of glucose and rCCL18 significantly increased Fn (*p* < 0.001), in comparison to high concentrations of glucose alone. HK-2 cells stimulated with glycated albumin consistently produced Fn and this did not alter following co-stimulation with rCCL18 or rMCP-1.

**Conclusion:**

This study demonstrates how stimulation with a specific chemokine CCL18 in high glucose upregulates the production of Fn from proximal tubuloepithelial cells. This may be relevant to the development of renal fibrosis in diabetic nephropathy

## Background

Diabetic nephropathy (DN) is an important complication of diabetes mellitus that despite current treatment often results in the development of chronic kidney disease and end stage kidney disease. The time course for progression is variable and has traditionally been associated with increasing proteinuria [1]; however, this marker may now be less useful as ACEi/ARB may cause reduction in proteinuria but not necessarily a halt in disease progression [2]. Glomerular and tubulointerstitial fibrosis are seen in renal biopsies of those with DN and may be a better marker of decline in renal function [3]. Understanding the mechanisms of fibrosis is therefore important. Fibronectin (Fn) is a protein found in the extracellular matrix and has a number of binding sites that may bind surfaces containing collagen and heparin. The production of Fn has been described in experimental DN as one of the extracellular matrices that contribute to the development of glomerulosclerosis [4]. Fn has also been reported to be increased in the glomeruli of patient and animal models of DN [5] and is an important marker of fibrosis. The effect of the diabetic milieu on Fn production by intrinsic renal cells is not fully understood.

Fibronectin production is upregulated in immortalised mouse podocytes stimulated with high glucose in-vitro, supporting the profibrotic effects of the diabetic environment [6–8]. There are reports of decreased Fn production when podocytes are stimulated with transforming growth factor-β (TGF-β) [9]; however, the effect of this in a diabetic environment is unclear. Podocytes treated with insulin appear to be protected from apoptosis via anti-angiotensin 2 mechanisms [10]. The protective effect of insulin on podocytes has been reported to be limited following stimulation with albumin that results in an increase in Fn production and apoptosis [10]. A recent study looking into the effects of toll-like receptor-4 in a mouse model of DN has reported an upregulation of TGF-β and Fn genes when mouse tubuloepithelial cells were cultured in high glucose [11].

Fn levels have been reported to increase in human proximal tubuloepithelial cells exposed to high glucose compared with normal glucose in-vitro [12]. This finding has been reversed using the compound Fasudil, a Rho-associated coiled-coil forming protein serine/threonine kinase(ROCK) inhibiting renal fibrosis. However, the effect of cytokines on Fn production in human proximal tubuloepithelial cells in diabetic conditions is unclear. Increasingly, studies suggest that proteinuria and loss of renal function correlate closely with the severity of underlying tubulointerstitial lesions [13]. Other studies report that tubulointerstitial cells play a role in epithelial-myofibroblast transdifferentiation (EMT) in DN [12]. In addition, Tervaert’s histopathological classification for DN reflects an appreciation of the role of the tubulointerstitium in DN [3]. We investigated the ability of HK-2 cells to produce Fn and whether this is affected by stimulation with high glucose or glycated albumin, as found in the diabetic milieu. We then examined the effect of costimulation with recombinant chemokines (CCL18, MCP-1) that have previously been detected in the urine of patients with DN.

There are numerous studies reporting the importance of inflammation in the progression and development of DN [14]. The diabetic milieu has been reported to induce the production of inflammatory molecules that stimulate signalling cascades facilitating migration of inflammatory cells [14–16]. Macrophage infiltration has been reported in the renal biopsies of patients with diabetic nephropathy (DN) [3, 17]. Cytokines can orchestrate the migration of inflammatory cells into renal tissues [14] with some pro-inflammatory cytokines such as tumour necrosis factor-α, interleukin-1 (IL-1), IL-6, IL-18 contributing to renal tubular damage and progression of DN [18–20].

A number of studies have reported an association between urinary chemokines/cytokines and the progression of DN, in particular CCN2/MCP-1 (monocyte chemoattractant protein-1) [21]. Urinary CTGF has been reported to correlate with the progression of DN [22]. Our group has previously reported C-C chemokine ligand 18 (CCL18/PARC) urinary levels to be raised in proteinuric diabetic patients compared to a non-diabetic proteinuric renal disease cohort [23]. Urinary cytokines may arise from intrinsic renal cells or infiltrating inflammatory cells.

This study examines the effect of the diabetic milieu on Fn production by HK-2 cells in-vitro. In addition, the effects of stimulation with recombinant CCL18 (rCCL18) or recombinant MCP-1 (rMCP-1) on Fn production from HK-2 cells are reported.

## Methods

### Materials

30 mM D-glucose was used as high glucose concentration for the following experiments, with Mannitol used as the osmotic control (26 mM +4 mM of D-glucose). Glycated albumin (A8301, Sigma, Gillingham, UK) was used at a concentration of 500 μg/ml +4 mM D-glucose, with physiological 4 mM D-glucose used as its control. The Fn ELISA was carried out using rabbit anti-human Fn polyclonal Ab for capture (F3648, Sigma, Gillingham, UK) and biotinylated murine anti-human Fn monoclonal Ab (F7387, Sigma, Gillingham, UK) for detection. Fn derived from human plasma was used as the antigen for the standard curve (range 1.95-2000 pg/ml) (F0895, Sigma, Gillingham, UK).

Recombinant human CCL18 (394-PA, R&D systems, Abingdon, UK) and MCP-1 (279-MC, R&D systems, Abingdon, UK) were reconstituted according to the manufacturer’s instructions. Following dose response experiments, HK-2 cells were stimulated with recombinant CCL18 or rMCP-1 at a concentration of 20 ng/ml for 48 h. Fn levels were subsequently measured from the cell supernatant using ELISA.

### Cell culture

HK-2 cells are an immortalised proximal tubuloepithelial cell line from normal adult human kidney that was a gift from Professor Roger Mason, Imperial College London, London, UK. Cells were grown at 37 °C in T75 tissue culture flasks until 70 % confluent and then 5x10^6^ cells were seeded into 6 well plates and maintained in keratinocyte media supplemented with bovine pituitary extract and epidermal growth factor (Life Technologies, GIBCO), 5% FCS, penicillin and streptomycin. Once the cells reached 70 % confluence in the 6 well plates, they were maintained for a further 24 h before their media was changed to RPMI 1640 supplemented with 1000U/ml penicillin, 100 g/ml streptomycin and 2 mM glutamine without serum.

Proximal tubuloepithelial cells are intrinsic renal cells that may be associated with release of cytokines in the urine. Pilot studies were performed using HK-2 cells stimulated with cytokines (Montero, unpublished data) previously identified in the urine of patients with DN [21, 23]. These studies suggested an interaction between HK-2 cells in the diabetic environment stimulated with rCCL18 or rMCP-1, hence informing the design of this study.

The glucose condition of choice (see above) was placed in each well alone (*n* = 6) or co-stimulated with rCCL18 (*n* = 6) or rMCP-1 (*n* = 6) for 48 h. Each condition was replicated in 6 wells for each experiment. Each experiment was repeated three times to ensure reproducible results. The plates were incubated and the supernatants were collected at 48 h.

### Analysis of samples

Supernatants were collected from the 6 well plates and centrifuged to remove cell debris. The supernatants were stored at -80 °C for later analysis. The [3-(4,5-dimethylthiazole-2,5-diphenyltetrazolium bromide], (MTT) assay (Sigma, UK) was performed on the 3 wells for each cultured condition to assess cell viability and read on the ELISA plate reader at 550 nm. Direct cell count was performed using Trypan blue exclusion assay (Sigma, UK).

### Enzyme-linked Immunoabsorbent Assay (ELISA)

The fibronectin in the cell culture supernatant samples was quantified by ELISA. Briefly Fn capture Ab was used to coat a 96 well Nunc plate overnight at 4 °C. The plate was washed three times and incubated in blocking solution for 1 h. The plate was washed thrice and the samples and standard applied, leaving the plate to incubate overnight at 4 °C. The plate was washed three times and incubated for 1 h with the detection Ab. The plate was washed and the Streptavidin, Substrate and Stop solution steps were as per standard ELISAs. The ELISA plate reader was used at 492 nm wavelength. Intra and inter-assay variability was 5.4 % and 8.7 %, respectively.

### Statistics

Graph Pad PRISM software (version 4) was used to perform data analysis. Fibronectin concentration was analysed using Kruskal-Wallis with Dunn’s post analysis for multiple comparisons. MTT assay and direct cell count with Trypan blue exclusion assay results were presented as mean ± SD and assessed using Analysis of variance with Bonferroni’s correction for multiple comparisons. *P* value < 0.05 compared with the control were considered as statistically significant.

## Results

### Diabetic milieu

#### Effect of high concentration of glucose on fibronectin production

**Fig. 1:**
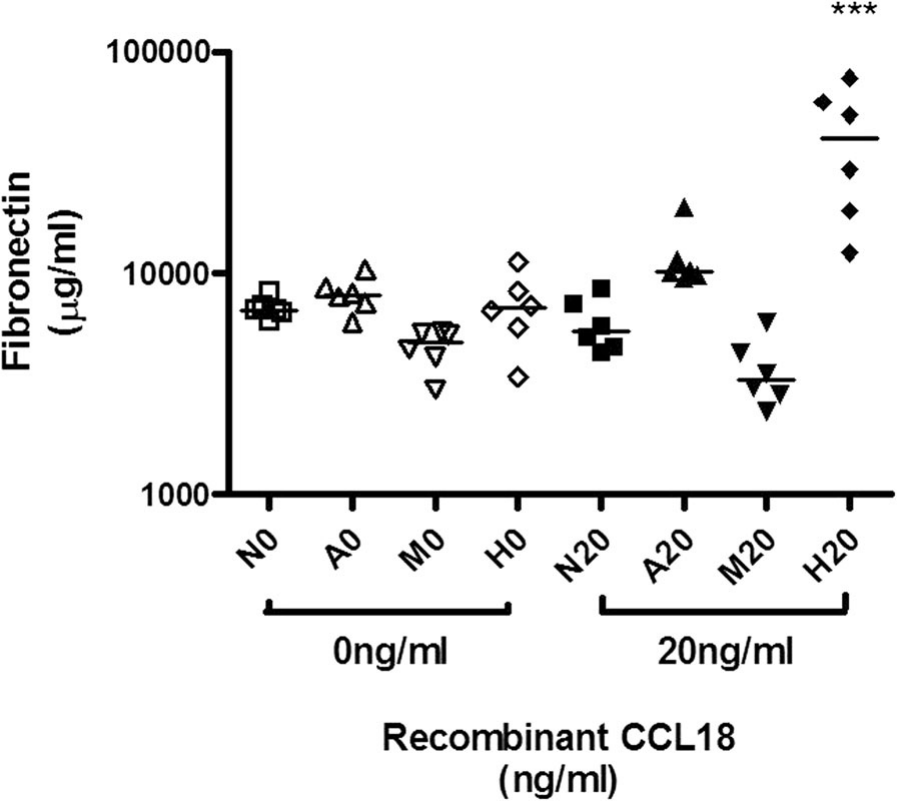
The production of fibronectin (Fn) by HK-2 cells stimulated with recombinant CCL18 for 48 h in a diabetic milieu. A significantly higher concentration of Fn was produced by HK-2 cells stimulated with recombinant CCL18 in high concentration of glucose in comparison to CCL18 only or high glucose concentration only. *P* < 0.001. Key: N0 = physiological glucose, A0 = glycated albumin, M0 = mannitol, H0 = high glucose, N20 = physiological glucose + 20 ng/ml CCL18, A20 = glycated albumin + 20 ng/ml CCL18, M20 = mannitol + 20 ng/ml CCL18, H20 = high glucose + 20 ng/ml CCL18

Fn was detected in the supernatant of HK-2 cells following 48 h stimulation with high glucose with similar levels seen in the osmotic control mannitol (median 6948 μg/ml (range 3414–11250) and median 4883 μg/ml (range 2971–5405), respectively, non-significant) (Fig. 1).

#### Effect of glycated albumin on fibronectin production

Fn was detected in the supernatant of HK-2 cells following 48 h stimulation with physiological glucose (4 mM D-glucose) at a median concentration of 6813 μg/ml (range 6112–8210) and when stimulated with glycated albumin in the presence of 4 mM D-glucose at median Fn levels of 7976 μg/ml (range 5998–10410). Differences were not significant (Fig. 1).

### Recombinant cytokines

#### Effect of co-stimulation with rCCL18 on fibronectin production

There was a significant rise in the levels of Fn in the supernatant of HK-2 cells following stimulation with rCCL18 in the presence of high concentrations of glucose compared to high concentrations of glucose only (*p* < 0.001) (Fig. 1). The Fn levels were not significantly raised in glycated albumin conditions with rCCL18, in comparison to glycated albumin only at 48 h.

#### Effect of co-stimulation with rMCP-1 on fibronectin production

**Fig. 2:**
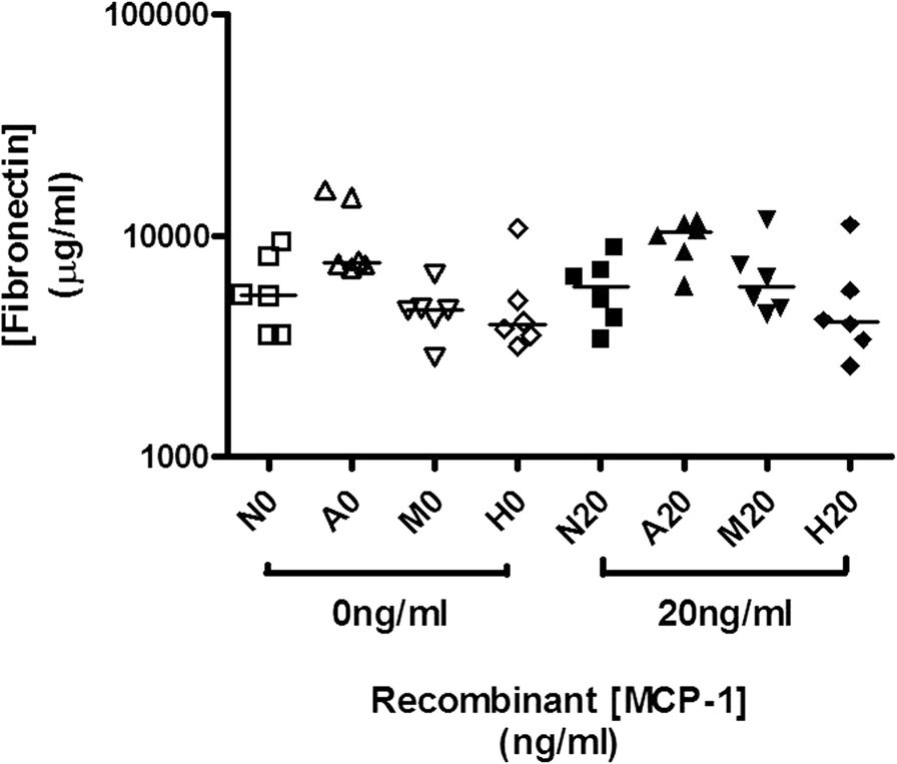
The production of fibronectin by HK-2 cells stimulated with recombinant MCP-1 for 48 h in a diabetic milieu. There were no any significant differences in Fn production in HK-2 cells stimulated under different conditions. Key: N0 = physiological glucose, A0 = glycated albumin, M0 = mannitol, H0 = high glucose, N20 = physiological glucose + 20 ng/ml MCP-1, A20 = glycated albumin + 20 ng/ml MCP-1, M20 = mannitol + 20 ng/ml MCP-1, H20 = high glucose + 20 ng/ml MCP-1

There were no significant differences in the Fn levels in the supernatant of HK-2 cells cultured with glycated albumin or high glucose when stimulated with rMCP-1 for 48 h, compared with those without additional rMCP-1 (Fig. 2).

### Cell viability assays

MTT assay showed an overall decrease in cell viability of HK-2 cells cultured in glycated albumin that did not alter following co-stimulation with cytokines. There were an increased number of surviving cells in high glucose conditions, but the difference did not reach statistical significance (Tables 1 and 2). We further investigated the number of viable cells in cells stimulated with high glucose concentration with or without recombinant stimulation with CCL18 using trypan blue exclusion assay. We found that the number of viable cells was not signficantly different between stimulation with high concentration of glucose only vs manitol osmolarity control and recombinant CCL18 vs high concentration of glucose and recombinant CCL18 (Table 2). The number of viable cells cultured with mannitol and normal glucose concentration was lower than in the other conditions.

**Table 1:** MTT assay of HK-2 cells in different experimental conditions and with or without co-stimulation with recombinant cytokines rCCL18 or rMCP-1 for 48 h

Condition with 0 ng/ml or 20 ng/ml of recombinant cytokine stimulation	MTT assay for HK-2 cells stimulated without or with rCCL18 Mean ± SD	MTT assay for HK-2 cells stimulated without or with rMCP-1 Mean ± SD
N0	0.664 ± 0.024	0.404 ± 0.072
A0	0.331 ± 0.040 **	0.107 ± 0.029 *
M0	0.692 ± 0.073	0.390 ± 0.005
H0	0.843 ± 0.131	0.603 ± 0.033
N20	0.633 ± 0.085	0.517 ± 0.059
A20	0.353 ± 0.042 **	0.263 ± 0.199
M20	0.590 ± 0.113	0.183 ± 0.008
H20	0.758 ± 0.057	0.365 ± 0.178

*Abbreviations*: *N* physiological glucose, *A* glycated albumin, *M* mannitol, *H* high glucose

Legend to Table 2: The results from two independent experiments are shown. MTT assay were carried out for 3 wells with each of the cell culture conditions. The results are presented as mean ± SD. The data were tested by Analysis of Variance with Bonferroni’s correction for multiple comparisons. **p < 0.01, * *p* < 0.05, in comparison to the cells culture in normal glucose concentration

**Table 2:** Number of viable HK-2 cells (assessed by trypan blue exclusion assay), in different experimental conditions with or without co-stimulation recombinant CCL18 for 48 h

Condition with 0 ng/ml or 20 ng/ml of recombinant cytokine stimulation	Number of live cells for HK-2 cells stimulated without or with rCCL18 (x1000 cells per well, Mean ± SD)
M0	288 ± 17 **
H0	467 ± 22
M20	420 ± 22
H20	437 ± 43

*Abbreviations*: M0 = mannitol control + normal glucose, H0 = high glucose, M20 = mannitol + normal glucose + 20 ng/ml CCL18, H20 = high glucose + 20 ng/ml CCL18. The number of viable cells were assessed by direct cell count with trypan blue exclusion assay. The results are presented as mean ± SD. The data were tested by Analysis of Variance with Bonferroni’s correction for multiple comparisons. The number of viable cells were lower in cells culture in the M0 group (**p < 0.01), in comparison to the cells culture in other conditions. There was no significant differences in cell counts between H0, M20 and H20 groups

In conclusion, there is increased production of fibronectin in HK2 cells stimulated with combination of recombinant CCL18 and high glucose concentration, in comparison to high concentration of glucose only or recombinant CCL18 and mannitol control with normal glucose concentration. The number of viable HK2 cells were not significantly different when assessed by both MTT and trypan blue exclusion assays.

## Discussion

This study demonstrates that HK-2 cells in high glucose co-stimulated with rCCL18 in-vitro increase the production of Fn compared to a high concentration of glucose only. This result cannot be explained by differences in cell viability, with no significant difference seen by direct cell count using trypan blue exclusion assay or MTT assay. Increased Fn production was not seen following co-stimulation with rMCP-1 or with glycated albumin. The MTT assay showed an overall decrease in cell viability of HK-2 cells in glycated albumin that did not alter with co-stimulation with rCCL18 or rMCP-1. Previous studies have reported an increase in Fn production between physiological and high glucose that was not seen in our present study [12]. Gu and colleagues measured fibronectin following stimulation with a much higher concentration (60 mmol/L) of D-glucose on HK-2 cells for a long duration of 72 h, and thus the difference in time course may explain the discrepancy between our findings.

It is established in DN that excess glucose binds to free amino acids on tissue proteins or those in the circulation. The non-enzymatic glycosylation that occurs, results in the formation of Advanced Glycated End Products (AGEPs). Initially, these bonds are reversible and attach to the matrix components of the glomerulus or the GBM; later, these bonds become irreversible. The AGEPs can accumulate throughout the body’s tissues as they are unable to be excreted due to the glomerular damage. Increasing amounts of AGEPs in tissues may result in microvascular complications [24]. Nitric oxide (NO) concentrations are reduced in a dose-dependent manner with the formation of AGEPs, exacerbating hypertension [25]. The advanced products then interfere with signal transduction. This may occur by changing soluble signals such as cytokines, hormones and free radicals. AGEPs are also known to be profibrotic in humans, interacting with the renin angiotensin system, cell signalling and RAGE, all disrupting the cellular matrix [26].

We therefore used glycated albumin as an advanced glycated protein to stimulate HK-2 cells to determine whether this would induce a different response in Fn production and whether this could be affected by stimulation with recombinant chemokines. The CCL18 effect on Fn production observed in high glucose was not seen with glycated albumin. These results suggested that the synergistic effects of CCL18 and high glucose concentration on Fn production are not dependent on stimulation of RAGE, or that the effect was limited by reduced viability of glycated albumin stimulation. The toxicity of albumin on proximal tubuloepithelial cells has previously been reported [27]. It appears that CCL18 or MCP-1, are unable to induce mechanisms to prevent the toxic effect of glycated albumin on these cells [28].

CCL18 is not produced by HK-2 cells (Montero unpublished data); however, these cells respond to CCL18 in a profibrotic manner. CCL18 has previously been described to be released from alternatively activated macrophages that contribute to idiopathic lung fibrosis [29, 30]. In addition, serum CCL18 has been described to be raised in patients with lung fibrosis [31], scleroderma [32], inflammatory diseases such as rheumatoid arthritis [33], acute lymphoblastic leukaemia [34] and gastric malignancies [35]. Inflammatory cells have been described to infiltrate the renal parenchyma in diabetic nephropathy in humans and also in rodent models [27, 36]. The ability of HK-2 cells to respond to CCL18 to alter the production of Fn in a high glucose environment, but not in physiological glucose, illustrates that the effect of a cytokine also depends on the metabolic environment.

Fn is an important matrix component that is detected in the glomeruli and tubulointerstitium of the kidneys during development and later in adulthood [37–39]. Proximal tubuloepithelial cells have previously been shown to produce Fn through activation of ERK, p38 MAPK, PKCα and PKCβII signalling pathways [40]. Fn production may subsequently be increased further through its downstream activation of TGF-β1, which leads to further fibrosis. Cell associated Fn has been reported to regulate cell-matrix interactions thereby stabilising the ECM [41, 42]. In contrast, soluble Fn has more of an immunoregulatory role in murine tubuloepithelial cells, causing an increase in MCP-1 and macrophage inflammatory protein-2 via ERK and NF-kB pathways [43]. The ability of HK-2 cells to produce variable amounts of Fn determined by their local environment illustrates the complexity of signalling pathways that may be induced or inhibited in association with different factors [44]. Further studies are needed to determine the underlying cell signalling pathways that result in an increase in Fn seen in HK-2 cells co-stimulated with CCL18 in high concentrations of glucose.

Urinary MCP-1 has been reported to be increased in patients with microalbuminuria and macroalbuminuria, with the latter predictive of progression of DN [21, 45]. This study showed no change in the production of Fn in HK-2 cells in a diabetic milieu with rMCP-1 co-stimulation. This would suggest that local production of MCP-1 may contribute to inflammatory cell recruitment but not directly to changes in the extracellular matrix, unlike CCL18.

This study has certain limitations. We examined protein levels of Fn but not mRNA levels in HK-2 cells, and thus could not distinguish between the different spliced forms of Fn (EDA+/-). Future experiments can be conducted to determine the predominant form of Fn produced. The findings from in-vitro studies may not be representative of in-vivo studies; however, there is no rodent equivalent of CCL18.

Despite the limitations of in-vitro cell culture, this work allows some insight into the pathogenesis of DN. The cytokines examined have been reported to be raised in patients with DN and albuminuria. The synergistic effect of CCL18 with high glucose to increase the production of Fn in HK-2 cells illustrates the importance and complexity of the roles of inflammatory, metabolic and fibrotic pathways. Understanding how these may interlink will help in the prevention of the progression of DN.

## Conclusion

This study demonstrates that stimulation with chemokine CCL18 in high glucose upregulates the production of Fn from proximal tubuloepithelial cells. This may explain fibrotic changes in diabetic nephropathy. Further studies are required to elucidate the cell signalling events that induce these effects and correlate them with clinical findings.
